# Meetings of Societies

**Published:** 1904-03

**Authors:** 


### MEETINGS OF SOCIETIES.

### Bristol Medico-Chirurgical Society.

*December 9th, 1903.*

Mr. J. Paul Bush, C.M.G., President, in the Chair.

Mr. T. J. Tonkin read a paper on **Leprosy in Jamaica,** illustrated by lantern slides. [*Vide* p. 1.] The paper was discussed by Mr. Paul Bush, Dr. Theodore Fisher, Dr. Michell Clarke, Dr. Steele, Dr. Cave and Dr. Alexander, and Mr. Tonkin replied.

Mr. G. Munro Smith showed **specimens from a case of gastro-jejunostomy.** The patient died on the fifth day, although the *post-mortem* examination showed that the union of the stomach and jejunum had been perfect and no leaking had occurred. In spite of good mechanical results these cases sometimes succumbed, and the present case was an example of this, death following the operation from toxæmia and shock.

Mr. F. LACE read notes on a case of intestinal obstruction due to a hair-ball, and showed the specimen. Obstruction had lasted two days, and had been accompanied by incessant biliary and stercoraceous vomiting and great abdominal pain: the latter interfered with the examination of the abdomen, but a swelling was found in the right iliac fossa. The patient's mother was convinced that a hair-ball had formed, and one was found situated in the jejunum. It was removed through a two-inch incision into the bowel. The subsequent history had been uneventful. As a child the patient had been observed to suck her hair. She had undergone treatment for gastric ulcers when fourteen years of age. Soltau Fenwick had reported twenty-four cases of the affection, all except two of which had been fatal, and two of them had been fatal from intestinal obstruction.

---

*January 13th, 1904.*

Mr. J. PAUL BUSH, C.M.G., President, in the Chair.

Dr. H. ELWIN HARRIS showed **a case of congenital absence of the left auricle and external auditory canal with facial paralysis.** In addition, the palate was much higher and moved less perfectly on the left than on the right side. No operative treatment was contemplated.—Mr. J. LACY FIRTH alluded to the fact that in these cases of congenital atresia auris the deafness was of the middle ear type, and that though operations had been tried for the purpose of improving the hearing, they had been found to be useless.

Mr. H. F. MOLE showed the following cases and specimens: (*a*) Patients on whom the "**radical mastoid** operation," or "ossiculectomy," had been performed for chronic suppuration in the middle ear; (*b*) A case of **choledochotomy**, with specimen: (*c*) A case of tumour of the nasal septum, with specimen—the whole of the cartilaginous septum had been removed; (*d*) Skiagrams of an unusual dislocation of the ankle—the foot had been displaced upwards between the tibia and fibula.—Mr. J. LACY FIRTH referred to the different degrees in which the mastoid antrum had been rendered accessible in the radical mastoid cases shown. He liked best to get a very free permanent access to that cavity as a result of the operation, and on the whole had found Ballance's first described plastic procedure the most satisfactory for the purpose. Some cases were disappointing from long persistence of the otorrhœa even after the operation.—Mr. W. ROGER WILLIAMS discussed septal tumours of the nose, and asked as to the nature of the specimen shown. He referred to Moore's case of sarcoma of the septum. —Mr. MOLE in replying said that the antrum, if small, filled up after the radical operation. He also liked a good wide opening leading to the antrum. The method by splitting the meatus backwards sometimes led to trouble in the after-treatment. There had been no recurrence of the septal tumour. Sections showed spindle-celled tissue.

Dr. J. M. FORTESCUE-BRICKDALE showed: (*a*) A **specimen of accessory gall-bladder**; and (*b*) The **brain of a child showing simple meningitis and considerable dilatation of the ventricles.** The accessory gall-bladder had the form of a small cyst lying in a groove in the anterior surface of the liver. It had been discovered at a *post-mortem* in a child of two years of age, and had not given rise to any symptoms or signs during life. The brain was shown because it well illustrated dilatation of the ventricles. The symptoms had not been definite, except that of head-retraction. Both ears of the patient had contained pus and streptococci.

Mr. JAMES TAYLOR showed a **skiagram** from a case of **scurvy rickets.** The child, ten months old, had been reared on a proprietary food from the first fortnight after birth. There were the signs of rickets and the lower third of the right thigh was enlarged by a firm swelling surrounding the shaft of the femur. The skiagram showed displacement of the lower epiphysis of the femur.

Dr. E. W. HEY GROVES read notes on and showed a specimen from a case of **tubal gestation** producing severe internal hemorrhage without rupture. [See p. 46]. Mr. W. ROGER WILLIAMS remarked that the case illustrated the dependence of extra-uterine gestation upon anomalies of structure.

Dr. W. KENNETH WILLS read a paper on the present position of **radiography in therapeutics.** [See p. 39.]—Dr. A. J. HARRISON alluded to the good results which occurred after severe reaction, and he emphasized the fact that the rays often greatly relieved pain.—Dr. P. WATSON WILLIAMS asked if it would not hasten the treatment in lupus cases if the parts were first scraped and then treated with the rays. He also inquired as to the possibility of treating lupus involving nasal and other cavities, especially the lachrymal duct. Mr. JAMES TAYLOR said that the use of the rays was empirical, for it was not known what would heal and what would not. He had found the rays useless in epithelioma. He thought that rodent ulcers with a dry surface reacted better than those with a moist surface. The relief of pain produced was often so great, *e.g.* in cancer *en cuirasse*, and this fact alone was sufficient to justify the use of the rays.—Mr. PAUL BUSH protested against allowing a case of cancer which was operable to be first treated by X-rays. Valuable time was thus wasted.—Dr. K. WILLS in replying said that his objection to a burn, as opposed to a deep erythematous dermatitis, was that it wasted much time and caused considerable pain. He considered that the same results appeared in the end without a burn. The effect of the rays in diminishing pain was better than that of medicinal anodynes, for the patient retained the full use of his faculties. The relief was only temporary, and the application of the rays had to be repeated in about forty-eight hours. The objection to first scraping lupus was that it produced scar tissue, which scatters the rays as much as ground glass diffuses ordinary light rays. Lupus in deep cavities, as far as his experience went, had not done very well. One case of nasal lupus was giving great promise, when the patient had to leave Bristol. With regard to treating cancer, he had only drawn attention to the views expressed by certain writers on the subject. In this country the general consensus of opinion was adverse to even trying the treatment in operable cases.

Dr. Theodore Fisher read a paper on **hematemesis associated with small white kidneys.** The speaker reported four cases illustrating this association. In each careful search failed to lead to the discovery in the stomach, œsophagus or duodenum of a source for the hemorrhage. In each the liver was free from cirrhosis. The possible explanation of the hematemesis in these cases was discussed, and the speaker thought that it was most probably in some way connected with the excretion of toxines by the stomach.

---

*February 10th, 1904.*

Mr. J. Paul Bush, C.M.G., President, in the Chair.

Dr. H. Waldo showed the chest viscera from a case of **hæmoptysis due to a bronchial lymphatic gland sloughing into the œsophagus.** The patient, a boy, had had a cough since Christmas, and had been admitted to the Bristol Royal Infirmary a few days ago, after having had several attacks of hæmoptysis. The temperature was raised, and there had been coarse râles at the left base. Death resulted from hæmoptysis. At the *post-mortem* the left lung was found to sink in water, and a large sloughing lymphatic gland to have made its way into the œsophagus. —Mr. Munro Smith asked where the hemorrhage had been produced.—Dr. Shingleton Smith said the case was interesting as illustrating a possible but rare cause of hæmoptysis. It was known that in rare cases the sloughing out of calcareous particles in the lungs, produced by old phthisis, might cause hæmoptysis.—Dr. Waldo replied that the blood had come from the trachea, and was due to erosion of vessels in the root of the lung.

Dr. F. H. Edgeworth showed a case of **cretinism.** It was the only form of imbecility which could be improved by treatment. The child was four years old, but had the weight of a child of three months and the intelligence of one a month old. It had supra-clavicular deposits of fat, a snub nose, pink cheeks, prominent abdomen with a small umbilical hernia, short limbs, but with the breadth of the epiphyses normal, and slight bowing of the tibiæ. The head was longer and not so square as usual in such cases. Skiagrams showed ossification of the diaphyses only, and the peculiar breadth of the extremities of the diaphyses. No enlargement of the thyroid gland existed. In achondroplasia the same bone changes were present, but without imbecility, and possibly, in this disease, there had been during intra-uterine life a temporary suspension of the activity of the thyroid gland. In treatment these cretins should be given as much thyroid extract as they could stand. It was unwise to push the dosage so as to accelerate the pulse above 110, and in the case he was showing two grains a day was the maximum dose which could be given. With three grains a day the pulse became 116.—Dr. D. A. Alexander alluded to the possible relationship of osteomalacia with imperfect thyroid activity, and to the treatment of eclampsia with immense doses of thyroid extract.—Dr. Michell Clarke recommended the use of the Liquor Thyroidei of the British Pharmacopœia rather than of dried extracts.—Mr. J. Lacy Firth mentioned a case of cretinism he had treated with thyroid extract in which there had been an enormous goitre present, the largest he had ever seen. Numerous adenomata could be felt in the goitre. A marvellous reduction in size had resulted from the treatment, and the adenomata had become freely movable in the rest of the glandular substance. Thyroid adenomata were not apparently amenable to this treatment, but they were commonly associated with parenchymatous enlargement. In the case mentioned he attributed the diminution in size to the effect produced on this associated parenchymatous enlargement.—Dr. Edgeworth replied that a drawback to the use of Liquor Thyroidei was that it decomposed on keeping. The bone changes in osteomalacia were quite different to those described above. He thought the action of the drug in eclampsia might be due to hastened metabolism with the production of more urea. The urea acted as a diuretic, and the same effect might possibly be produced more directly by administering urea.

Dr. J. Michell Clarke showed: (*a*) A **case of lesion of the cauda equina**; (*b*) A **case of lesion of the lumbar cord.**

Dr. Walter Swayne showed a **calcareous mass removed from the broad ligament.** The patient, a woman of sixty, had suffered from pains in the lower abdomen, for which treatment had proved unavailing. A hard mass was felt per rectum to the left of the uterus. On opening the abdomen the mass was found to be adherent to the intestines and omentum and to be calcareous. The uterus was atrophied, and presented two small fibroids at the top, but had no connection with the mass. Through its coverings a grating on pressure had been yielded by the swelling. He thought it possible that the mass was a calcified abscess in the broad ligament, the result of typhoid fever thirty years before.—Mr. H. F. Mole asked if the mass might not be a calcified fibroid of the broad ligament.—Dr. Alexander alluded to a case of recurrent attacks of severe abdominal pain he had seen, which had been at first thought to be appendicitis; later, as a positive Widal reaction had been given, it was thought to be typhoid fever. Ultimately it proved to be a case of intestinal obstruction from a gall-stone. He asked if it was possible that the typhoidal symptoms and signs could have been due to the gall-stone leading to the liberation of bacilli. There was a previous history of typhoid fever.—Mr. F. Lace thought the tumour was a calcified fibroid of the broad ligament. He had a larger specimen of the same kind removed from a broad ligament. In that case there had been other calcified fibroids present.—Dr. Kenneth Wills asked if the density of the adhesions had been such as would be likely to be met with around a calcified abscess.—Dr. Swayne thought it was quite possible that the tumour had been a calcified fibroid. The peculiar elasticity with grating of the tumour on palpation had not suggested that view at the time of operation. The mass had not been buried in dense adhesions posteriorly.

Mr. G. Munro Smith showed: (*a*) A skiagram showing **foreign bodies in the hand.** Five bodies were shown in the skiagram, one being a brooch-pin. The patient, a woman, had come complaining of having a needle in the forefinger. It seemed certain that she had voluntarily pushed these bodies into the hand. (*b*) A specimen of a **scrotal tumour.** The patient had had a hydrocele tapped some time previously. Probably then a hæmatocele had formed, and the hard swelling shown had subsequently formed. Microscopical examination favoured the view that the tumour was inflammatory. It surrounded the testis, which was normal.

Mr. A. W. Prichard read notes of **three cases of vesical calculus** he had recently operated upon by lithotomy, and showed the specimens. He alluded to the fact that the disease seemed to be much less frequent in the district than in former times. Possibly this was the result of better feeding. Also stones were now more frequently removed from the kidney.

Mr. J. Dacre read notes on two unusual cases of **pneumothorax.** Case 1 was that of a young man of twenty. On bending down one morning he suddenly felt a pain in the chest on the right side. He went to Trowbridge the same morning, but on arriving there the pain had become so intense that he had to return home. He was then found to look pale and ill and to have quick and shallow breathing. The right chest was found to be hyper-resonant from top to bottom, and the heart displaced an inch to the left. He was sent to bed. During the first few days the dyspnœa increased, and the apex beat became displaced further to the left. The chest was tapped and air allowed to bubble out through carbolic lotion. Faintness soon came on, and the tube had to be removed. Later the chest was tapped again; but again the faintness came on, the heart having come back one inch nearer the normal position. He was then treated by perfect rest, and in three weeks to a month recovered completely. His chest had remained normal since, a period of two or three years, and there had been no previous history of pulmonary disease. Case 2 was that of a healthy youth of eighteen. After a hearty supper he went to the theatre, and when there felt suddenly sick, went out, and vomited freely. He returned to his seat, but again became faint and had to be given brandy. He complained of great epigastric pain. Next morning the speaker found him looking pale, but regarded the attack as having been one of colic. On the following day he was worse, and the signs of pneumothorax had developed, and also a friction sound synchronous with the heart beats. There was no rise of temperature. The patient was kept at perfect rest for a month, and at the end of eight weeks was quite well. The speaker ascertained that the youth had had a ledger-lifting contest with other clerks fourteen days previously, and had felt a sudden pain in the chest then. Perhaps he had ruptured a lung vesicle, weakened from the above accident, when vomiting, and this led to the friction and pneumothorax developing. He thought the cases showed that a favourable prognosis was warrantable in such cases in healthy individuals. The question of insurance claims under an accident policy came up in one of the cases. The company had decided that they were not liable, but awarded him compensation.—Dr. Michell Clarke thought the first patient must have had an old tubercular lesion. He only recollected seeing two cases of pneumothorax without phthisis.—Dr. Waldo said the current view was that there must be a previous lesion. He mentioned the case of a young lady who suddenly developed the affection when dancing. Six weeks' rest had cured her.—Dr. Shingleton Smith felt satisfied that he had met with pneumothorax without there being any evidence of the previous existence of a pulmonary lesion, and he thought it was not right to assume that such cases had all a tubercular origin.—Dr. Markham Skerritt alluded to the production of pneumothorax from traumatism and in whooping-cough. He recollected having treated one case, soon after he came to Bristol, by tapping time after time. And only the other day he met the patient, who was quite well still. He had met with other cases of the kind. His own practice was to advise tapping on the same principle as he would advise it in other effusions, viz., if there were pressure symptoms. Aspiration was not required. There was not the fear of the lung getting fixed down by adhesions in these cases as in some other effusions.—Dr. Edgeworth suggested that both patients should be examined with the X-rays to see if the diaphragm was immobile on the affected side. This was a sign sometimes given of pulmonary disease in the absence of all other signs.

J. LACY FIRTH.
H. F. MOLE (*Hon. Sec.*).

### Local Medical Notes.

